# Draft genome sequence of *Mycobacterium rufum* JS14^T^, a polycyclic-aromatic-hydrocarbon-degrading bacterium from petroleum-contaminated soil in Hawaii

**DOI:** 10.1186/s40793-016-0167-5

**Published:** 2016-08-02

**Authors:** Yunyoung Kwak, Qing X. Li, Jae-Ho Shin

**Affiliations:** 1 School of Applied Biosciences, College of Agriculture and Life Sciences, Kyungpook National University, Daegu, 702-701 Republic of Korea; 2 Department of Molecular Biosciences and Bioengineering, University of Hawaii, Honolulu, HI 96822 USA

**Keywords:** *Mycobacterium*, Polycyclic aromatic hydrocarbon, Biodegradation

## Abstract

*Mycobacterium rufum* JS14^T^ (=ATCC BAA-1377^T^, CIP 109273^T^, JCM 16372^T^, DSM 45406^T^), a type strain of the species *Mycobacterium rufum* sp. . belonging to the family *Mycobacteriaceae*, was isolated from polycyclic aromatic hydrocarbon (PAH)-contaminated soil in Hilo (HI, USA) because it harbors the capability of degrading PAH. Here, we describe the first genome sequence of strain JS14^T^, with brief phenotypic characteristics. The genome is composed of 6,176,413 bp with 69.25 % G + C content and contains 5810 protein-coding genes with 54 RNA genes. The genome information on *M. rufum* JS14^T^ will provide a better understanding of the complexity of bacterial catabolic pathways for degradation of specific chemicals.

## Introduction

Polycyclic aromatic hydrocarbons, defined as organic molecules consisting of two or more fused aromatic rings in linear, angular, or cluster arrangement, mostly result from coke production, petroleum refining, fossil fuel combustion, and waste incineration [1]. Although the physical and chemical properties of PAHs vary depending on the number of rings, the characteristics such as hydrophobicity, recalcitrance, and mutagenic and carcinogenic potentials have been considered the main factors for the toxic effects on environmental ecosystems and human beings [1, 2].

For removal of PAHs from contaminated environments, the bioremediation process based on microbial activities has attracted interest and has been actively studied [3]. Various bacteria, such as *Sphingomonas* spp., *Pseudomonas* spp., *Rhodococcus* spp., *Burkholderia* spp., and *Mycobacterium* spp., have been investigated regarding whether they can metabolize PAHs. In particular, several *Mycobacterium* species have been reported to effectively degrade high-molecular-weight PAHs [4, 5]. Moreover, genomic studies on these bacterial species have contributed to the understanding of whole regulatory mechanisms of bacterial PAH degradation, for example for *M. vanbaalenii* PYR-1 [6], *M. gilvum* Spyr1 [7], and *M. gilvum* PYR-GCK [8] as well as the most recently reported *M. aromaticivorans* JS19b1^T^ [9].

*M. rufum* JS14^T^ (=ATCC BAA-1377^T^, CIP 109273^T^, JCM 16372^T^, DSM 45406^T^) is the type strain of the species *Mycobacterium rufum* sp. nov. [10]. This bacterium was isolated from petroleum-contaminated soil at a former oil gasification company site in Hilo (HI, USA). The bacterium was identified because of PAH degradation activities, especially toward a four-ring-fused compound, fluoranthene [11]. Although the PAH-degrading ability has been demonstrated through metabolic and proteomic assays [12], genetic studies on the whole bacterial system with a PAH degradation pathway have not been conducted. Here, we present a brief summary of the characteristics of this strain and a genetic description of its genome sequence.

## Organism information

### Classification and features

**Fig. 1:**
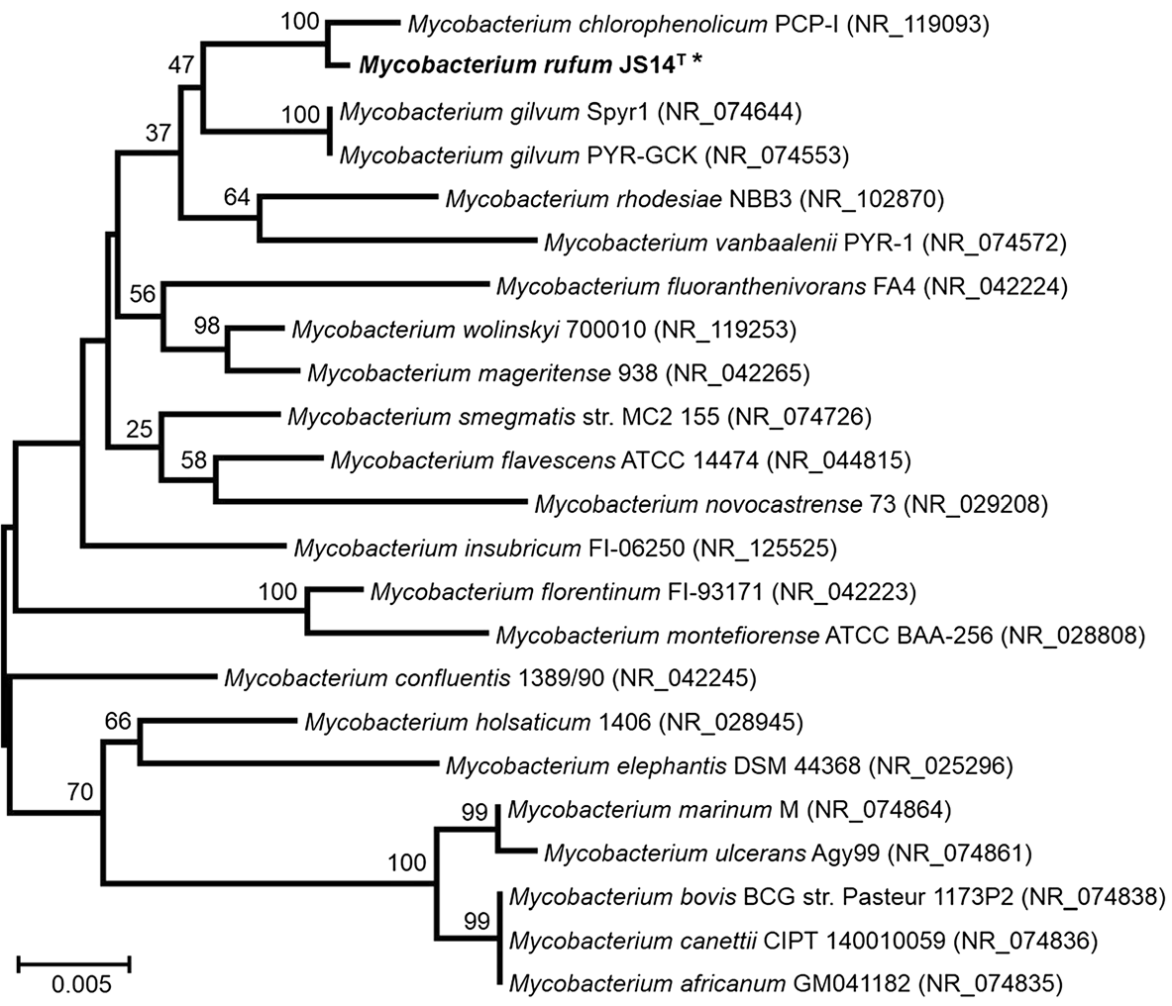
A neighbor-joining phylogenetic tree depicting the position of *M. rufum* JS14^T^ [10] (shown in boldface with an asterisk) relative to the other species within the genus *Mycobacterium*. In this genus, species carrying the full length of 16S rRNA gene sequence were selected from the NCBI database [45]. The collected nucleotide sequences were aligned using ClustalW [46], and the phylogenetic tree was constructed using software MEGA version 6 [47] by the neighbor-joining method with 1000 bootstrap replicates [18]. The generated bootstrap values for each species are presented at the nodes, and the scale bar indicates 0.005 nucleotide changes per nucleotide position. The strains under study and their corresponding GenBank accession numbers for 16S rRNA genes are as follows: *M. chlorophenolicum* PCP-I [14, 15] (NR_119093); *M. gilvum* Spyr1 [37, 48] (NR_074644); *M. gilvum* PYR-GCK [37, 48] (NR_074553); *M. rhodesiae* NBB3 [49] (NR_102870); *M. vanbaalenii* PYR-1 [16] (NR_074572); *M. fluoranthenivorans* FA4 [17, 50] (NR_042224); *M. wolinskyi* 700010 [51] (NR_119253); *M. mageritense* 938 [52] (NR_042265); *M. smegmatis* str. MC2 155 [37, 53] (NR_074726); *M. flavescens* ATCC 14474 [37, 54] (NR_044815); *M. novocastrense* 73 [55] (NR_029208); *M. insubricum* FI-06250 [56] (NR_125525); *M. florentinum* FI-93171 [57] (NR_042223); *M. montefiorense* ATCC BAA-256 [58, 59] (NR_028808); *M. confluentis* 1389/90 [60] (NR_042245); *M. holsaticum* 1406 [61] (NR_028945); *M. elephantis* DSM 44368 [62] (NR_025296); *M. marinum* M [37, 63] (NR_074864); *M. ulcerans* Agy99 [37, 64] (NR_074861); *M. bovis* BCG str. Pasteur 1173P2 [37, 65] (NR_074838); *M. canettii* CIPT 140010059 [66] (NR_074836); *M. africanum* GM041182 [37, 67] (NR_074835)

The 16S ribosomal RNA gene sequence of *M. rufum* JS14^T^ was compared with those from other *Mycobacterium* species using the BLAST software of NCBI [13]. The highest similarity was found with *M. chlorophenolicum* PCP-1 (99 % identity) [14, 15] followed by *M. gilvum* Spyr1 (99 % identity) [7], *M. gilvum* PYR-GCK (99 % identity) [8], *M. vanbaalenii* PYR-1 (98 % identity) [16], and *M. fluoranthenivorans* FA4T (97 % identity) [17]. Species identified by the BLAST search and represented by full-length 16S rRNA gene sequences were included in the phylogenetic analysis. The phylogenetic tree was generated by the neighbor-joining method [18], and bootstrapping was set to 1000 times for random replicate selections. The consensus phylogenetic neighborhood of *M. rufum* JS14^T^ within the genus *Mycobacterium* is shown in Fig. 1.

**Fig. 2:**
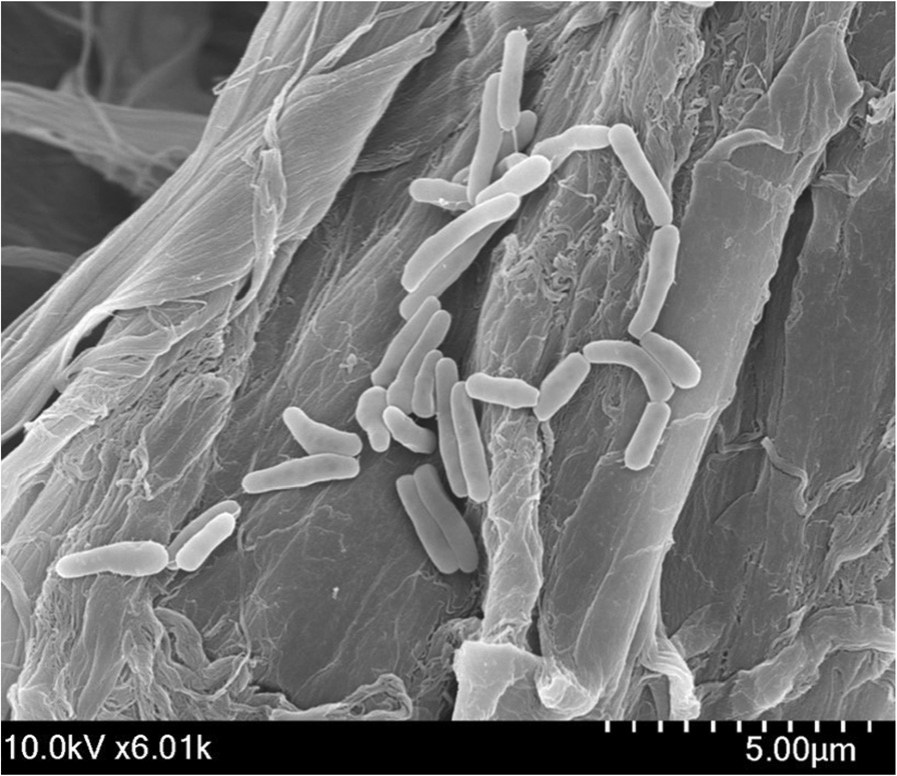
A scanning electron micrograph of *M. rufum* JS14^T^. The image was taken using a Field Emission Scanning Electron Microscope (SU8220; Hitachi, Japan) at an operating voltage of 10.0 kV. The scale bar represents 5.0 μm

*M. rufum* JS14^T^ is a non-motile, aerobic, Gram-positive bacterium belonging to the family *Mycobacteriaceae* [10]. The cell shape is medium-to-long thin rods, and cell size is approximately 1.0–2.0 μm in length with the width of 0.4–0.6 μm as shown in Fig. 2. Generally, large, round, raised, smooth orange-pigmented colonies form within 7 days [10]. As one of the rapidly growing members of the genus *Mycobacterium*, the strain grows optimally at 28 °C, reduces nitrate, but does not tolerate salinity (over 2.5 % NaCl, w/v) [10]. Strain JS14^T^ shows positive reactions in tests for catalase, *α*-glucosidase, aesculin hydrolysis, and urease, but negative reactions regarding *β*-glucuronidase, *β*-galactosidase, *N*-acetyl-*β*-glucosaminidase, gelatin hydrolysis, alkaline phosphatase, and pyrrolidonyl arylamidase activities [10]. Substrate oxidation was noticed for Tween 40, Tween 80, D-gluconic acid, D-glucose, D-fructose, D-xylose, D-mannose, D-psicose, trehalose, dextrin, glycogen, and D-mannitol, but not for α-/β-cyclodextrin, D-galactose, α-D-lactose, maltose, sucrose, mannan, or maltotriose [10]. When cultured in the minimal medium (per liter: 8.8 g of Na_2_HPO_4_°2H_2_O, 3.0 g of KH_2_PO_4_, 1.0 g of NH_4_Cl, 0.5 g of NaCl, 1.0 mL of 1 M MgSO_4_, and 2.5 mL of a trace element solution [per liter: 23 mg of MnCl_2_°2H_2_O, 30 mg of MnCl_4_∙H_2_O, 31 mg of H_3_BO_3_, 36 mg of CoCl_2_°6H_2_O, 10 mg of CuCl_2_°2H_2_O, 20 mg of NiCl_2_°6H_2_O, 30 mg of Na_2_MoO_4_°2H_2_O, and 50 mg of ZnCl_2_]) [11] supplemented with the four-aromatic ring-fused PAH compound fluoranthene (final concentration of 40 mg/L), *M. rufum* JS14^T^ showed an effective degrading action on the added compound by utilizing it completely during 10 days as a sole source of carbon and energy [11].

#### Chemotaxonomic data

The main cellular fatty acids of *M. rufum* JS14^T^ are C18:1ω9c (36.72 %), C16:0 (26.24 %), C16:1ω7c + C16:1ω6c (9.40 %), C17:1ω7c (8.44 %), C14:0 (5.27 %), C18:0 (3.14 %), and C17:0 (1.94 %), respectively [10]. The profile of whole-cell fatty acids showed a pattern similar to that of the other representative of *Mycobacterium* species [10, 19–21]. The strain showed bright red color under a microscope after acid-fast staining. A gas chromatogram of fatty acid methyl esters from the transmethylated cells of *M. rufum* JS14^T^ revealed a major C24:0 peak and a trace of a C22:0 peak. The general characteristics of the strain are summarized in Table 1.

**Table 1:** Classification and general features of *M. rufum* JS14^T^ [22]

MIGS ID	Property	Term	Evidence code^a^
	Classification	Domain *Bacteria*	TAS [33]
		Phylum *Actinobacteria*	TAS [34]
		Class *Actinobacteria*	TAS [35]
		Order *Actinomycetales*	TAS [36–38]
		Family *Mycobacteriaceae*	TAS [37–39]
		Genus *Mycobacterium*	TAS [37, 40, 41]
		Species *Mycobacterium rufum*	TAS [37, 39]
		(Type) strain: JS14^T^ (=ATCC BAA-1377^T^, CIP 109273^T^, JCM 16372^T^, DSM 45406^T^)	TAS [10]
	Gram stain	Positive: weak uptake of Gram stain	TAS [10]
	Cell shape	Medium to long thin rods	TAS [10]
	Colony pigmentation	Orange	TAS [10]
	Motility	Non-motile	TAS [10]
	Sporulation	Not reported	NAS
	Temperature range	Mesophile	NAS
	Optimum temperature	28 °C	TAS [10]
	pH range; Optimum	7.0–8.0; 7.5	NAS
	Carbon source	Fluoranthene, glucose, fructose, mannitol, trehalose, xylose, others	TAS [10–12]
	Energy source	Fluoranthene	TAS [11, 12]
MIGS-6	Habitat	Soil	TAS [10]
MIGS-6.3	Salinity	Not tolerant salinity (2.5–5.0 % NaCl, w/v)	TAS [10]
MIGS-22	Oxygen requirement	Aerobic	TAS [10]
MIGS-15	Biotic relationships	Free living	NAS
MIGS-14	Pathogenicity	None	NAS
MIGS-4	Geographic location	Hawaii, United States	TAS [10]
MIGS-5	Sample collection	February, 2003	NAS
MIGS-4.1	Latitude	19° 49′ 20″ N	TAS [11]
MIGS-4.2	Longitude	155° 05′ 01″ W	TAS [11]
MIGS-4.3	Depth	Not reported	
MIGS-4.4	Altitude	Not reported	

^a^ *Evidence codes.* IDA: Inferred from Direct Assay; TAS: Traceable Author Statement (i.e., a direct report exists in the literature); NAS: Non-traceable Author Statement (i.e., not directly observed for the living, isolated sample, but based on a generally accepted property for the species, or anecdotal evidence). These evidence codes are from the Gene Ontology project [42]

## Genome sequencing information

### Genome project history

Strain *M. rufum* JS14^T^ was selected for sequencing because of its effective ability to degrade PAH, as a model organism for a recalcitrant organic-pollutant-degrading bacterium. The genome sequencing was performed in September, 2014, and the Whole Genome Shotgun project was deposited in the DDBJ/EMBL/GenBank databases under the accession number JROA00000000. The version described in this study is the first version, labeled JROA00000000.1. The sequencing project information and its association with the Minimum Information about a Genome Sequence version 2.0 compliance [22] are described in Table 2.

**Table 2:** Project information

MIGS ID	Property	Term
MIGS-31	Finishing quality	Draft
MIGS-28	Libraries used	20 kb SMRT-bell library
MIGS-29	Sequencing platforms	PacBio RS II
MIGS-31.2	Fold coverage	113.03×
MIGS-30	Assemblers	RS HGAP Assembly Protocol [24] in SMRT analysis pipeline v.2.2.0
MIGS-32	Gene-calling method	NCBI Prokaryotic Genome Annotation Pipeline [43]; GeneMarkS+ [44]
	Locus Tag	EU78
	INSDC ID	JROA00000000
	GenBank Date of Release	October 2, 2014
	GOLD ID	Gi0074119
	BIOPROJECT	PRJNA247390
MIGS-13	Source Material Identifier	ATCC BAA-1377^T^, CIP 109273^T^, JCM 16372^T^, DSM 45406^T^
	Project relevance	Environmental

### Growth conditions and genomic DNA preparation

*M. rufum* JS14^T^ from Deutsche Sammlung von Mikroorganismen und Zellkulturen GmbH (strain accession number DSM 45406^T^) was used for preparation of genomic DNA. The strain was cultured aerobically in a 250-mL Erlenmeyer flask containing 50 mL of tryptic soy broth (Difco Laboratories Inc., Detroit, MI), on a rotary shaker at 200 rpm and 30 °C. Genomic DNA was isolated from 50 mL of culture using the QIAamp® DNA Mini Kit (Qiagen, Valencia, CA) following the standard protocol recommended by the manufacturer. The quantity and purity of the extracted genomic DNA were assessed with a Picodrop Microliter UV/Vis Spectrophotometer (Thermo Fisher Scientific Inc., Waltham, MA) and Qubit® 2.0 Fluorometer (Fisher Scientific Inc.), respectively. Finally, a DNA concentration of 780.0 ng/μL and OD _260_/OD _280_ of 1.87 was determined.

### Genome sequencing and assembly

The genome of *M. rufum* JS14^T^ was sequenced using the single-molecule real-time DNA sequencing platform on the Pacific Biosciences RS II sequencer with P5 polymerase - C3 sequencing chemistry (Pacific Biosciences, Menlo Park, CA) [23]. A 20-kb insert SMRT-bell library was prepared from the sheared genomic DNA and loaded onto two SMRT cells. During the single 180-min run-time, 1,020,750,498 read bases were generated with 300,584 reads. Reads of less than 100 bp or with low accuracy (below 0.8) were removed. In total, 111,515 reads produced 823,795,879 bases with a read quality of 0.831.

All post-filtered reads were assembled *de novo* using the RS hierarchical genome assembly process, version 3.3 in SMRT analysis software, version 2.2.0 (Pacific Biosciences) [24] and resulted in 4 contigs corresponding to 4 scaffolds, with 113.03-fold coverage. The maximal contig length and N50 contig length had the same size of 5,760,162 bp. The whole genome was found to be 6,176,413 bp long.

### Genome annotation

The protein-coding sequences were predicted by Prokaryotic Genome Annotation Pipeline, version 2.8, on the NCBI website (rev. 447580) [25]. Additional gene prediction and functional annotation were performed in the Rapid Annotation using Subsystems Technology server [26] and Integrated Microbial Genomes-Expert Review pipeline [27], respectively.

## Genome properties

**Fig. 3:**
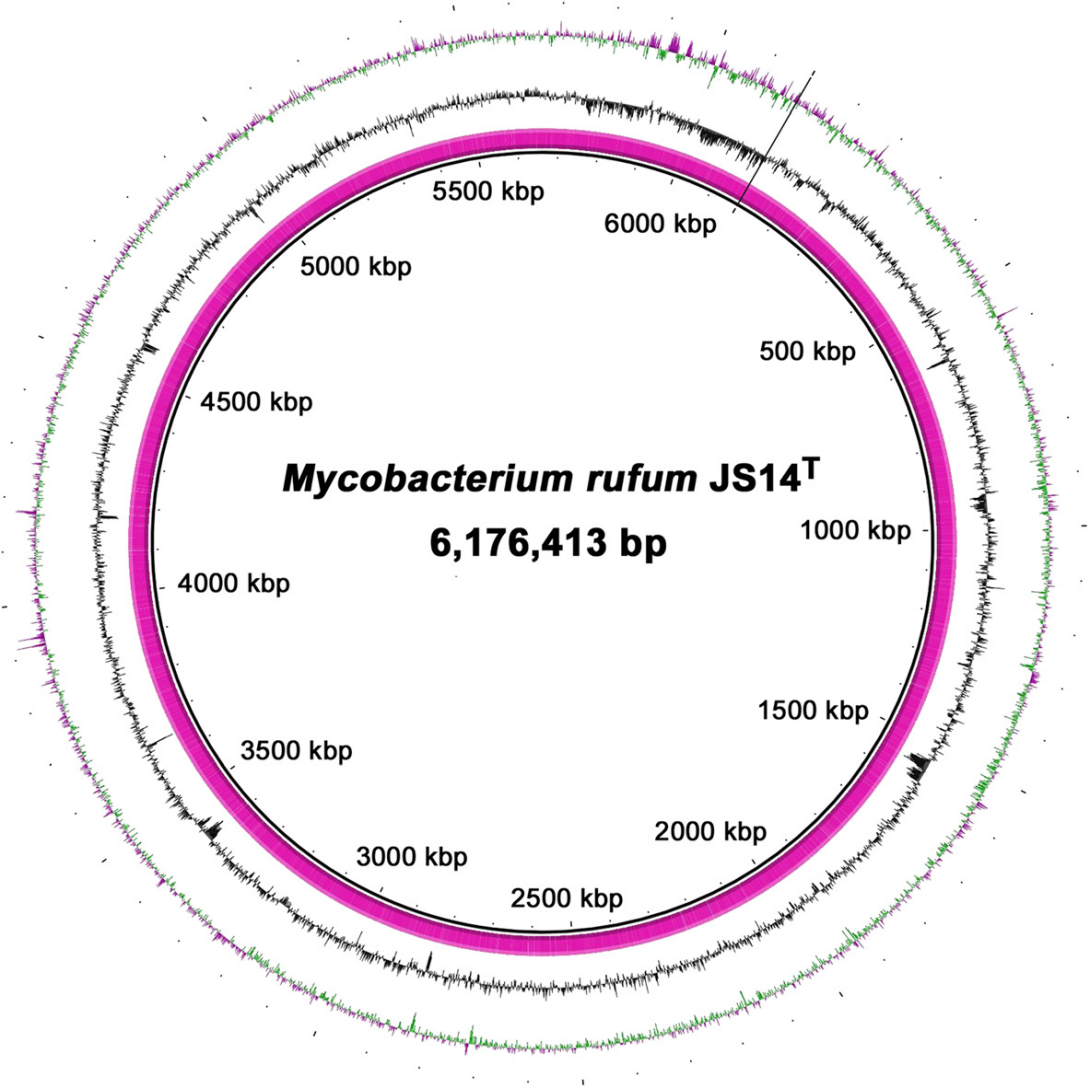
A graphical circular map of the *M. rufum* JS14^T^ genome. The circular map was generated using the BLAST Ring Image Generator software [68]. From the inner circle to the outer circle: Genetic regions; GC content (black), and GC skew (purple/green), respectively

The genome size of *M. rufum* JS14^T^ was found to be 6,176,413 bp with the average G + C content of 69.25 %. The genome was predicted to contain a total of 5864 genes, which include 5810 protein-coding genes with 54 RNA genes (6 rRNAs, 47 tRNAs, and 1 ncRNA). Of these, 4498 genes were assigned to putative functions, and 3669 genes (approximately 62.57 %) were assigned to the COG functional categories. The genome statistics are presented in Table 3 and Fig. 3, respectively. The gene distribution within the COG functional categories is presented in Table 4.

**Table 3:** Genome statistics

Attribute	Value	% of Total
Genome size (bp)	6,176,413	100.00
DNA coding (bp)	5,622,516	91.03
DNA G + C (bp)	4,277,025	69.25
DNA scaffolds	4	100.00
Total genes	5864	100.00
Protein-coding genes	5810	99.08
RNA genes	54	0.92
Pseudogenes	367	6.26
Genes in internal clusters	944	16.10
Genes with function prediction	4498	76.71
Genes assigned to COGs	3669	62.57
Genes with Pfam domains	4544	77.49
Genes with signal peptides	314	5.35
Genes with transmembrane helices	1227	20.92
CRISPR repeats	0	0.00

**Table 4:** Numbers of genes associated with general COG functional categories

Code	Value	% age	Description
J	181	4.25	Translation, ribosomal structure and biogenesis
A	1	0.02	RNA processing and modification
K	353	8.29	Transcription
L	118	2.77	Replication, recombination and repair
B	0	0.00	Chromatin structure and dynamics
D	32	0.75	Cell cycle control, cell division, chromosome partitioning
V	98	2.30	Defense mechanisms
T	173	4.06	Signal transduction mechanisms
M	210	4.93	Cell wall/membrane/envelope biogenesis
N	12	0.28	Cell motility
U	22	0.52	Intracellular trafficking, secretion, and vesicular transport
O	142	3.34	Post-translational modification, protein turnover, chaperones
C	312	7.33	Energy production and conversion
G	245	5.76	Carbohydrate transport and metabolism
E	333	7.82	Amino acid transport and metabolism
F	89	2.09	Nucleotide transport and metabolism
H	266	6.25	Coenzyme transport and metabolism
I	422	9.91	Lipid transport and metabolism
P	224	5.26	Inorganic ion transport and metabolism
Q	264	6.20	Secondary metabolites biosynthesis, transport and catabolism
R	516	12.12	General function prediction only
S	209	4.91	Function unknown
W	2	0.05	Extracellular structures
X	33	0.78	Mobilome: prophages, transposons
-	2195	37.43	Not in COGs

The total is based on the total number of protein coding genes in the annotated genome

## Insights from the genome sequence

Regarding the specific degradation capability toward the four-aromatic-ring-fused compound, fluoranthene [10–12], the genome of *M. rufum* JS14^T^ was found to contain corresponding genes encoding proteins for the aromatic-compound degradation.

Generally, it is known that an initial step of the bacterial degradation of PAHs is mainly catalyzed by multicomponent dioxygenases that produce dihydrodiols [28, 29]. In the genome, multiple genes encoding various dioxygenases such as aromatic-ring-hydroxylating dioxygenase (EU78_28655, 28730, 29130), extradiol dioxygenase (EU78_24090, 26390), protocatechuate 3,4-dioxygenase alpha subunit (EU78_29035), protocatechuate 3,4-dioxygenase beta subunit (EU78_29030), phthalate 3,4-dioxygenase ferredoxin reductase subunit (EU78_29090), and extradiol ring-cleavage dioxygenase (EU78_16970, 28720) were predicted. In addition, the genes coding for such enzymes as cytochrome P450 (EU78_02320, 09230, 14085, 14465, 20055, 26160), methyltransferase (EU78_01005), flavin-dependent oxidoreductase (EU78_19900), and 3,4-dihydroxyphthalate decarboxylase (EU78_28715) were also identified as functional genes on the Kyoto Encyclopedia of Genes and Genomes map [30] for the PAH degradation. Nonetheless, when compared with the complete genome sequences of PAH-degrading organisms [6–9], several genes coding for representative functional enzymes with relevance to PAH degradation such as *nidA* (PAH dioxygenase large subunit), *nidB* (PAH dioxygenase small subunit), *phtAa* (phthalate 3,4-dioxygenase alpha subunit), *phtAb* (phthalate 3,4-dioxygenase beta subunit), *phtB* (phthalate 3,4-cis-dihydrodiol dehydrogenase), *phdE* (cis-3,4-dihydrophenanthrene-3,4,-diol dehydrogenase), and *phdK* (2-formylbenzoate dehydrogenase) were not identified (shown in Table 5).

**Table 5:** Comparison of the functional gene counts in the function profile of genome sequences

Function ID	Name	*M. van* ^a^	*M. gil* GCK^a^	*M. gil* Sp1^a^	*M. aro* ^b^	*M. ruf* ^b^
KO:K00448	protocatechuate 3,4-dioxygenase, alpha subunit [EC:1.13.11.3] (*pcaG*)	1	1	1	1	3
KO:K00449	protocatechuate 3,4-dioxygenase, beta subunit [EC:1.13.11.3] (*pcaH*)	1	1	1	1	2
KO:K18253	phthalate 3,4-dioxygenase ferredoxin subunit (*phtAc*)	0	2	1	2	1
KO:K18254	phthalate 3,4-dioxygenase ferredoxin reductase subunit [EC:1.18.1.3] (*phtAd*)	1	2	1	0	1
KO:K00517	E1.14.-.- (cytochrome P450)	12	10	10	5	6
KO:K18256	3,4-dihydroxyphthalate decarboxylase [EC:4.1.1.69] (*phtC*)	1	2	1	0	1
KO:K11943	PAH dioxygenase large subunit [EC:1.13.11.-] (*nidA*)	1	2	1	1	0
KO:K11944	PAH dioxygenase small subunit [EC:1.13.11.-] (*nidB*)	2	4	2	4	0
KO:K11948	1-hydroxy-2-naphthoate dioxygenase [EC:1.13.11.38] (*phdI*)	1	2	1	0	0
KO:K11949	4-(2-carboxyphenyl)-2-oxobut-3-enoate aldolase [EC:4.1.2.34] (*phdJ*)	1	2	1	1	0
KO:K18251	phthalate 3,4-dioxygenase alpha subunit [EC:1.14.12.-] (*phtAa*)	1	2	1	0	0
KO:K18252	phthalate 3,4-dioxygenase beta subunit [EC:1.14.12.-] (*phtAb*)	1	2	1	1	0
KO:K18255	phthalate 3,4-cis-dihydrodiol dehydrogenase [EC:1.3.1.-] (*phtB*)	1	2	1	1	0
KO:K18257	cis-3,4-dihydrophenanthrene-3,4-diol dehydrogenase [EC:1.3.1.49] (*phdE*)	1	2	1	1	0
KO:K18275	2-formylbenzoate dehydrogenase [EC:1.2.1.78] (*phdK*)	1	1	1	1	0

Comparison of the selected five genome sequences was conducted using function profile categories in the IMG-ER pipeline [27], and the genome sequences analyzed are as follows: *M. van*, *M. vanbaalenii* PYR-1 (IMG Genome ID 639633044) [6]; *M. gil* GCK, *M. gilvum* PYR-GCK (IMG Genome ID 640427122) [8]; *M. gil* Sp1, *M. gilvum* Spyr1 IMG Genome ID 649633070) [7]; *M. aro, M. aromaticivorans* JS19b1 (whole Genome Sequencing) (IMG Genome ID 2558309009) [9]; *M. ruf, M. rufum* JS14 (whole Genome Sequencing) (IMG Genome ID 2593339261)

Reported sequencing status for the individual genome set: ^a^ Complete genome sequence; ^b^ Draft whole-genome sequence

Generally, research on bacteria degrading PAHs holds great promise for biotechnological applications to decontamination of pollutants [10]. In this regard, understanding of PAH degradation by indigenous microbes is important for evaluation of ecological effects of these microbes [31]. On Hawaiian islands, PAH contamination has occurred through various activities such as the petroleum industry, waste incineration, and fossil fuel combustion, even via natural causes such as volcanic activity [10]. *Mycobacterium* is a well-known genus capable of mineralizing PAHs [12]. Considering the Hawaiian delicate island ecosystem, several native bacteria belonging to the genus *Mycobacterium* were isolated, *M. rufum* JS14^T^ is one of them [10].

One of native isolates from the petroleum-contaminated Hawaiian soil in Hilo (HI, USA), *M. aromaticivorans* JS19b1^T^ [10], is known to have rapid degrading capabilities toward various PAHs such as fluorene, phenanthrene, pyrene, and fluoranthene [10, 11, 29]. Similarly, *M. rufum* JS14^T^ was found as an effective degrader of a four-aromatic-ring-fused compound, fluoranthene, not showing degrading capacity toward other high-molecular-weight PAHs (e.g., pyrene, benzo[*a*]pyrene) or toward low-molecular-weight PAHs (e.g.*,* fluorene, phenanthrene) [11, 12]. The gene annotation profiles for the genome of *M. rufum* JS14^T^ may provide important clues to the identity of the whole metabolic pathway for fluoranthene degradation. Just as a recent study on the functional pan-genome analysis of the genus *Mycobacterium* capable of degrading PAHs [32], our data can also help to explain the complexity of bacterial catabolic pathways for degradation of specific chemicals, from the standpoint of microbial ecology.

## Conclusions

*M. rufum* JS14^T^ was isolated from PAH-contaminated soil of a former oil gasification company site in Hilo (HI, USA) and was designated as a novel species that was named *Mycobacterium rufum* (ru’fum. L. neut. adj. *rufum* ruddy or red, pertaining to the colony pigmentation of the type strain) [10]. In this study, we presented the genome sequence of the strain. This genetic information may provide new insights that will help to extend the application potential of bacterial bioremediation of various toxic compounds and to elucidate the features of metabolic degradation pathways for PAHs.

## Abbreviations

PAHs: polycyclic aromatic hydrocarbons
SMRT: single-molecule real-time
